# KNTC1 as a putative tumor oncogene in pancreatic cancer

**DOI:** 10.1007/s00432-022-04146-3

**Published:** 2022-07-19

**Authors:** Ling Liu, Hongwei Chen, Xinan Chen, Chenjie Yao, Weimin Shen, Changku Jia

**Affiliations:** 1 Department of Hepatopancreatobiliary Surgery, Affiliated Hangzhou First People’s Hospital, Zhejiang University School of Medicine, Huansha Road, NO.261, Hangzhou, 310006 China; 2 Zhejiang Provincial Research Center for Diagnosis and Treatment of Heapatobiliary Diseases, Hangzhou, China; 3 Department of Key Laboratory of Clinical Cancer Pharmacology and Toxicology Research of Zhejiang Province, Affiliated Hangzhou First People’s Hospital, Zhejiang University School of Medicine, Hangzhou, China

**Keywords:** Kinetochore-associated protein 1, Oncogene, Pancreatic cancer, Cell division cycle associated 8, Carcinogenesis

## Abstract

**Purpose:**

Recent studies have demonstrated that kinetochore-associated protein 1 (KNTC1) plays a significant role in the carcinogenesis of numerous types of cancer. This study aimed to explore the role and possible mechanisms of KNTC1 in the development of pancreatic cancer.

**Methods and results:**

We analyzed differentially expressed genes by RNA sequencing in three paired pancreatic cancer and para-cancerous tissue samples and found that the expression of KNTC1 was significantly upregulated in pancreatic cancer. A Cancer and Tumor Gene Map pan-analysis showed that high expression of KNTC1 was related to poor prognosis in 9499 tumor samples. With immunohistochemical staining, we found that the high expression of KNTC1 in pancreatic cancer was related to pathological grade and clinical prognosis. Similarly, RT-PCR results indicated that the expression of KNTC1 was higher in three groups of pancreatic cancer cell lines (BxPC-3, PANC-1, and SW1990) than in normal pancreatic ductal cells. We introduced lentivirus-mediated shRNA targeting KNTC1 into PANC-1 and SW1990 cells and found that KNTC1 knockdown significantly decreased cell growth and increased cell apoptosis compared to the control group cells. Bioinformatic analysis of the cell expression profile revealed that differential genes were mainly enriched in the cell cycle, mitosis, and STAT3 signaling pathways, and co-immunoprecipitation confirmed an interaction between KNTC1 and cell division cycle associated 8.

**Conclusions:**

KNTC1 could be linked to the pathophysiology of pancreatic cancer and may be an early diagnostic marker of cervical precancerous lesions.

## Introduction

Pancreatic cancer is one of the most intractable malignant tumors, with a high degree of malignancy, poor prognosis, and 5 year overall survival rate of ~ 9% (Neoptolemos et al. 2018; Pishvaian et al. 2020). GLOBOCAN2018 estimated that from 2018 to 2040, global incidence (+ 77.7% of 356,358 new cases) and mortality (+ 79.9% of 345,181 deaths) rates in pancreatic cancer will see a sharp upward trend1 (Bray et al. 2018). Although we have made substantial progress in the research of multimodal therapy and genetics related to pancreatic cancer, the exact molecular mechanisms remain unclear. A better understanding of the genes involved in tumor progression may allow for the development of novel treatment strategies to tackle this rapidly progressing disease (Heinrich and Lang 2017; Furukawa 2009; Qian et al. 2020; Wang et al. 2011).

Kinetochore-associated protein 1 (KNTC1) is an evolutionarily conserved subunit of the centromere protein complex that is essential for spindle assembly and chromosome segregation (Moudgil et al. 2015; Maria et al. 2019; Trivedi et al. 2019). Before all chromosomes in the spindle are arranged correctly, an inhibitory signal is triggered to prevent late cell division (Jefford and Irminger-Finger 2006; Xu et al. 2011; Dürrbaum and Storchová 2016; Sacristan et al. 2018). KNTC1 is a key component of mitotic checkpoints that can participate in a variety of mechanisms to ensure correct chromosome separation during mitosis and prevent cells from prematurely ceasing mitosis (Liu et al. 2019a; Wang et al. 2021; Li et al. 2016; Devarbhavi et al. 2021). Chromosome segregation and cell division play key roles in the process of cell growth and proliferation (Batty and Gerlich 2019; Nagano et al. 2017; Nasmyth 2002). The process of cell division depends on many evolutionarily conserved protein complexes and its regulation involves many specialized proteins (Fukagawa 2015; Canman and Cabernard 2018). Compared to normal tissues, a variety of proteins that regulate mitosis are overexpressed in human malignant tumors, some of which are oncogenes (Liu et al. 2019b; Schmit and Ahmad 2007). Targeted knockout of centromere scaffolds, widely expressed in various primary tumors, can induce apoptosis of human tumor cells in vitro and significantly hinder the growth of implanted tumors in vivo (Fuchigami et al. 2020; Allan et al. 2020). From these studies, we posit that centromere proteins can be used as potential biomarkers for the early diagnosis of cancer, and understanding the role of centromere proteins could aid the development of personalized treatments for human malignant tumors. KNTC1 is a differentially expressed gene or survival-related gene in a variety of human malignant tumors, including esophageal squamous cell carcinoma, hepatocellular carcinoma, neuroblastoma and gastric cancer (Liu et al. 2019a; Wang et al. 2021; Wolf et al. 2010). However, to the best of our knowledge, no previous reports have described the significance of KNTC1 in pancreatic cancer, and the specific molecular mechanisms involved remain obscure.

The present study evaluated the relationship between KNTC1 and pancreatic cancer using differential gene and pan-cancer analyses, and analyzed the expression of KNTC1 in pancreatic cancer cell lines. We found that KNTC1 was upregulated in pancreatic cancer and likely participates in its progression. Finally, genomic enrichment analysis (GSEA) was performed to clarify the role of KNTC1 in pancreatic cancer tumorigenesis and to infer the potential mechanism.

## Materials and methods

### Cell culture and human tissue pancreatic cancer

The pancreatic cancer cell lines SW1990, PANC-1, and BxPC-3 and normal pancreatic ductal epithelial cells HPDE were purchased from the cell bank of the Shanghai Branch of the Chinese Academy of Sciences (Shanghai, China). These cell lines were cultured in RPMI1640 medium supplemented with 10% FBS, 100 IU/ml penicillin at 37 ℃ with 5% CO2. Three paired human pancreatic and para-cancerous tissue samples were obtained from patients who underwent surgical resection at the Affiliated Hangzhou First People’s Hospital, Zhejiang University School of Medicine, from 2013 to 2015. Tissue samples were histopathologically and clinically diagnosed. The samples were snap-frozen in liquid nitrogen and stored at − 80 °C until use. This study was approved by the Ethics Committee of the Hangzhou First People’s Hospital, Zhejiang University School of Medicine, and informed consent was obtained from all of the patients.

### RNA isolation and identification of DEGs

Total RNA was isolated from tissues using an RNeasy kit (Qiagen, Hilden, Germany). The integrity of each RNA sample was confirmed by post-extraction denaturing (glyoxal) agarose gel electrophoresis. The three paired samples were analyzed using RNA-seq and then subjected to a differential gene analysis. We used the linear model based on empirical Bayesian distribution to calculate the significant difference level *p* value, and used the Benjamini–Hochberg method to correct the significant difference level (FDR). The screening criteria for genes with significant differences were as follows: change in expression level > 2 and FDR < 0.05.

### Gene ontology and kyoto encyclopedia of genes and genomes enrichment analysis

For functional enrichment analysis, all DEGs were mapped to terms in the GO databases, and the significantly enriched GO terms were searched for among the DEGs using *p* < 0.05 as the threshold. GO term analysis was classified into three subgroups: biological process (BP), cellular component (CC), and molecular function (MF). All the DEGs were mapped to the KEGG database and searched for significantly enriched KEGG pathways at *p* < 0.05.

### TCGA pan-caner analysis

The gene expression data and clinical information used in this study can be found on the Cancer and Tumor Gene Map (TCGA) database (https://tcgamurdata.nci.nih.Govpool/). The original sequencing data were standardized and screened, and 9499 tumor samples were collected, all of which were associated with KNTC1 gene expression data and complete clinical records. The relationship between KNTC1 expression and the total survival time of patients with pan-cancer was evaluated using an online tool. The total survival time was defined as the time from pan-cancer diagnosis to death.

### Immunohistochemical (IHC) staining

Paraffin-embedded tissue slides were deparaffinized in xylene, rehydrated through graded alcohol solutions, blocked in methanol containing 3% hydrogen peroxide for 30 min, and incubated with KNTC1 antibodies at 4 ℃ overnight. Slides were rinsed in PBS and incubated with secondary antibodies and peroxidase reagents at room temperature for 20 min. Finally, the slides were incubated with 3,3-diaminobenzidine solution at room temperature for 10 min and counterstained with hematoxylin. A scoring scale was used to evaluate the percentage of stained cells (0, < 10%; 1, 10–25%; 2, 25–50%; 3, 50–75%; 4, > 75%) and the staining intensity (0, negative; 1, low; 2, moderate; 3, strong). The overall staining scores were determined as the product of the frequency and intensity scores. An immunohistochemical score > 6 was defined as high expression and a score ≤ 6 was considered low expression.

### Reverse transcription-PCR

Total RNA was isolated from cell pellets using TRIzol (TransGen Biotech, Beijing, China) according to the manufacturer’s protocol. The concentration and purity of the total RNA were determined using a NanoDmp 2000 spectrophotometer (TransGen Biotech, Beijing, China). cDNA was synthesized by reverse transcription using a PrimeScript RT reagent Kit (Takara Bio, Beijing, China), and then amplified by SYBR Premix Ex Taq II (Takara Bio, Beijing, China). The experiment was performed according to product specifications. The specific primers used were: KNTC1, forward 5′-ACCTGAGTGTCGGTTCAAGAA-3′ and reverse 5′-ACCTGAGTGTCGGTTCAAGAA-3′; GAPDH, forward 5′-TGACTTCAACAGCGACACCCACAMUR-3′ and reverse 5′-CACCCTGTTGCTGTAGCCAAA-3′. RT-qPCR was performed with an initial denaturation step at 95 ℃ for 15 s, followed by cycles at 95 ℃ for 5 s and 60 ℃ for 30 s. The experimental results were analyzed using the 2-CT method.

### Cell transfection

The KNTC1-targeted short hairpin RNA (shRNA) sequence (5'-TGAGTTTATGGGATATTTA-3') was designed by Shanghai GeneChem (Shanghai, China). The shRNA sequences were synthesized and cloned into a pGCSIL‑green fluorescent protein (GFP) lentiviral vector by Shanghai GeneChem. The nonsense shRNA (5'-TTCTCCGAACGTGTCACGT-3'), which shared no homology with any known human genes, was generated as a negative control (NC) (Shanghai GeneChem). Recombinant lentiviral transduction pancreatic cancer cells were seeded into six‑well plates until cell confluence reached 70%, and were then infected with the KNTC1–shRNA–lentivirus or the NC lentivirus at a multiplicity of infection of 50. Transduction effects were determined by detecting GFP expression 24–72 h post‑transduction using fluorescence and light microscopy. Cells were harvested 72 h post-transduction, and the knockdown effect was confirmed with RT‑qPCR and western blot analysis.

### CCK8 analysis

Pancreatic cancer cells in the logarithmic growth phase were inoculated in 96-well plates at 4 × 103 cells per well and measured every 24 h for 4 d. We added 100 μL DMEM and 10 μL CCK8 (Dojindo Laboratories, Kumamoto, Japan) to each well 1 h before the termination of the experiment, reading the optical density (OD) at 450 nm with a BioTeK Synergy H1 plate reader (Winooski, VT). The cell growth curve was estimated for each group with cell growth time as the *x*-axis and the ratio of the absorbance difference between the experimental group and the blank control group to the absorbance value on the first day as the *y*-axis.

### Flow cytometry assays

Human pancreatic cancer cells in the logarithmic growth phase were cultured by flow cytometry and inoculated in six-well plates at 2 × 104 cells per well for 5 d. Apoptosis was detected using an annexin V-FITC/ propidium iodide (PI) Apoptosis Detection Kit (BD Biosciences, Palo Alto, CA, USA). The resuspended cells were stained with annexin V-FITC/propidium iodide (PI) according to the manufacturer’s instructions. Using PI as a fluorescent dye, the percentage of cells at different stages of division was determined by fluorescence-activated cell sorting. The cell suspension was then added to the prepared PI staining solution (500 μL) and incubated in the dark for 30 min. Finally, the cell suspension was analyzed by flow cytometry.

### Western blot analysis

Total cellular protein was extracted according to the manufacturer’s instructions. Protein concentration was measured using the Bradford protein assay (Bio-Rad, Hercules, CA). The membrane was separated by sodium dodecyl sulfate–polyacrylamide gel electrophoresis (SDS–PAGE), transferred to a polyvinylidene fluoride and PVDF membrane, and treated with a monoclonal antibody (goat anti-rabbit KNTC1, goat anti-rabbit CDCA8, and β-actin monoclonal antibody) overnight at 4 ℃. Then, membranes were incubated with the appropriate secondary antibody for 1 h at room temperature. Finally, Protein bands were detected using the chemiluminescence detection system (Millipore Corp., Billerica, MA).

### Co-immunoprecipitation

To extract total protein, we used 500 μg of a cleavage solution (50 mmol/L Tris–HCl [pH 7.4],150 mmol/L NaCl, 0.1% SDS, 1% NP, 0.5% sodium deoxycholate, 1% protease inhibitor) with agarose beads pre-washed at 4 ℃ for 1 h, which was incubated overnight with specific primary antibody or normal control IgG at 4 ℃. After capturing the antigen–antibody complex, the agarose beads were collected by centrifugation, rinsed with PBS 3 times, boiled with 125 μL of 5 × SDS buffer for 5 min, and the corresponding proteins were detected by routine western blotting.

### Statistical analysis

All of the experiments were repeated at least three times. All data were analyzed using SPSS version 19.0 (IBM SPSS, Chicago, IL, USA) and R version 4.1.2 (www.r-project.org). Two-tailed unpaired Student’s *t* tests were used to compare differences between the groups. The Chi-square test was used to analyze the relationship between KNTC1 expression and the corresponding clinicopathological characteristics. The survival curve was plotted using the Kaplan–Meier method and compared using a log-rank test. Differences were considered statistically significant at *p* < 0.05.

## Results

### The upregulated expression of KNTC1 in pancreatic cancer

**Fig. 1:**
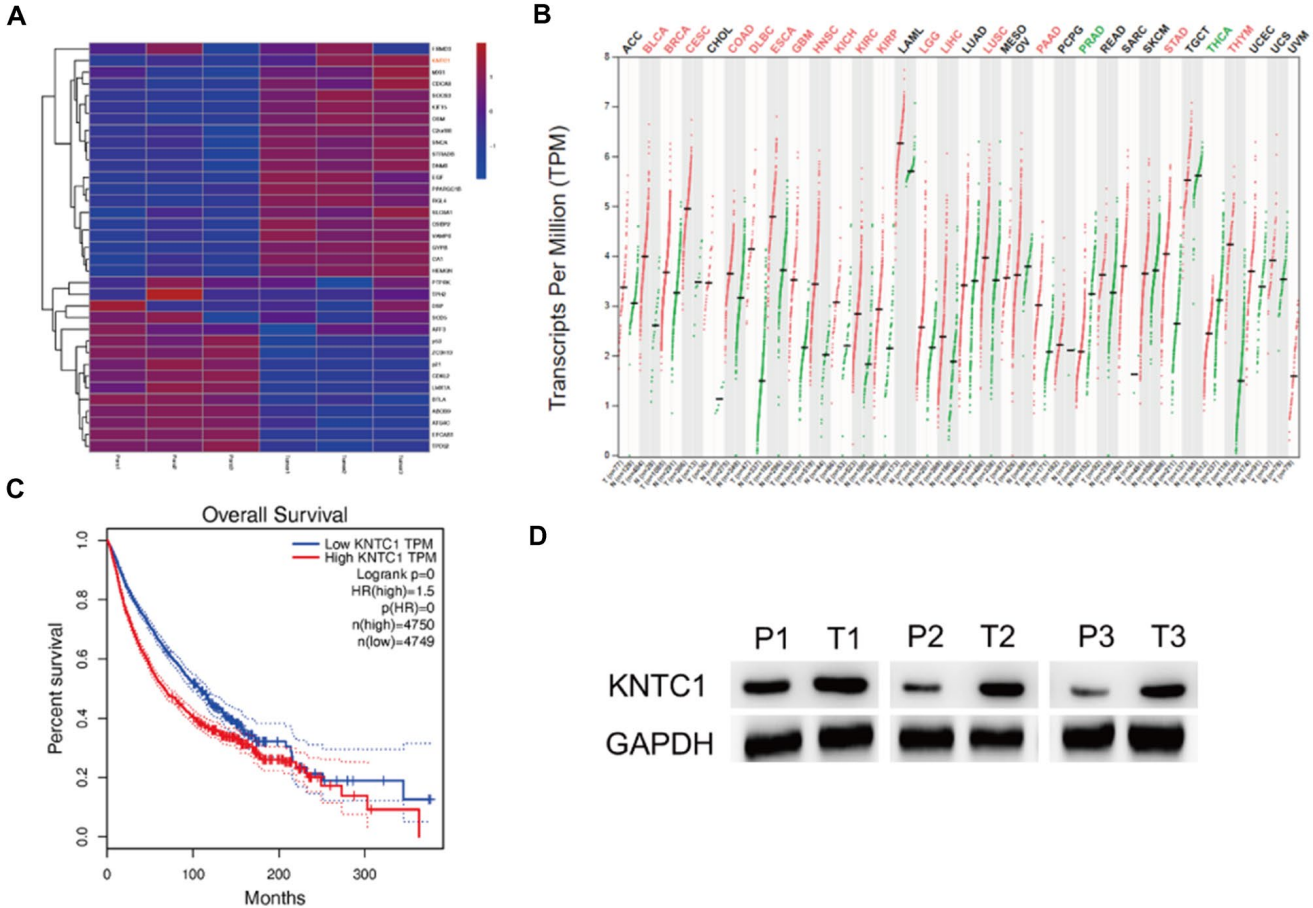
Gene expression profile and the protein expression level of KNTC1 in pancreatic cancer (**A**, **D**). TCGA pan-caner analysis of KNTC1 in most tumors (**B**, **C**)

We extracted total RNA from the three paired pancreatic cancer samples, which were subjected to RNA-seq and differential gene analyses. The expression of KNTC1 was significantly upregulated in pancreatic cancer. According to the western blot analysis, the protein expression level of KNTC1 was also upregulated in pancreatic cancer compared to the para-cancerous samples. We then examined the role of KNTC1 in tumors and found that the expression of KNTC1 was upregulated in most tumors and the prognosis of patients with high expression levels was poor. These results suggest that KNTC1 may act as an oncogene in pancreatic cancer (Fig. 1).

### The correlation between KNTC1 and clinicopathology in pancreatic cancer

**Fig. 2:**
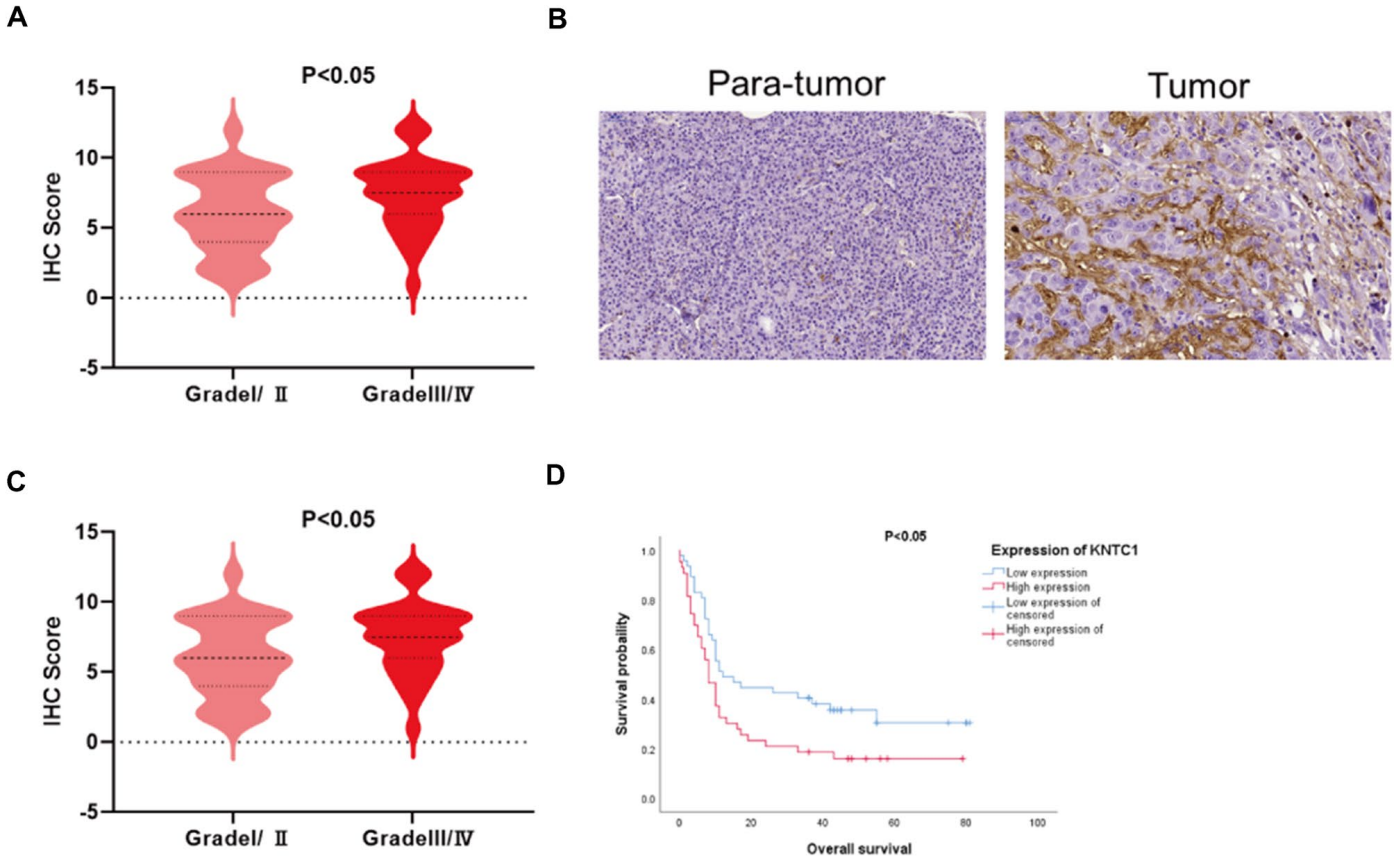
Immunohistochemical expression of KNTC1 in pancreatic cancer (**A**–**C**). The Kaplan–Meier survival curve and log-rank test showed a strong negative correlation between KNTC1 expression and overall survival (OS) (**D**)

We investigated the relationship between KNTC1 expression and clinicopathological features of pancreatic cancer. Through immunohistochemical detection of the clinical samples, the expression of KNTC1 in tumor tissues of patients with pancreatic cancer was found to be significantly upregulated and related to pathological grade and overall survival time. The Kaplan–Meier survival curve and log-rank test showed a strong negative correlation between KNTC1 expression and overall survival (OS) (Fig. 2). Thus, high KNTC1 expression was associated with high malignancy and poor prognosis in pancreatic cancer.

### Knockdown of KNTC1 inhibited cell proliferation and blocked the cell cycle in the G2 phase

**Fig. 3:**
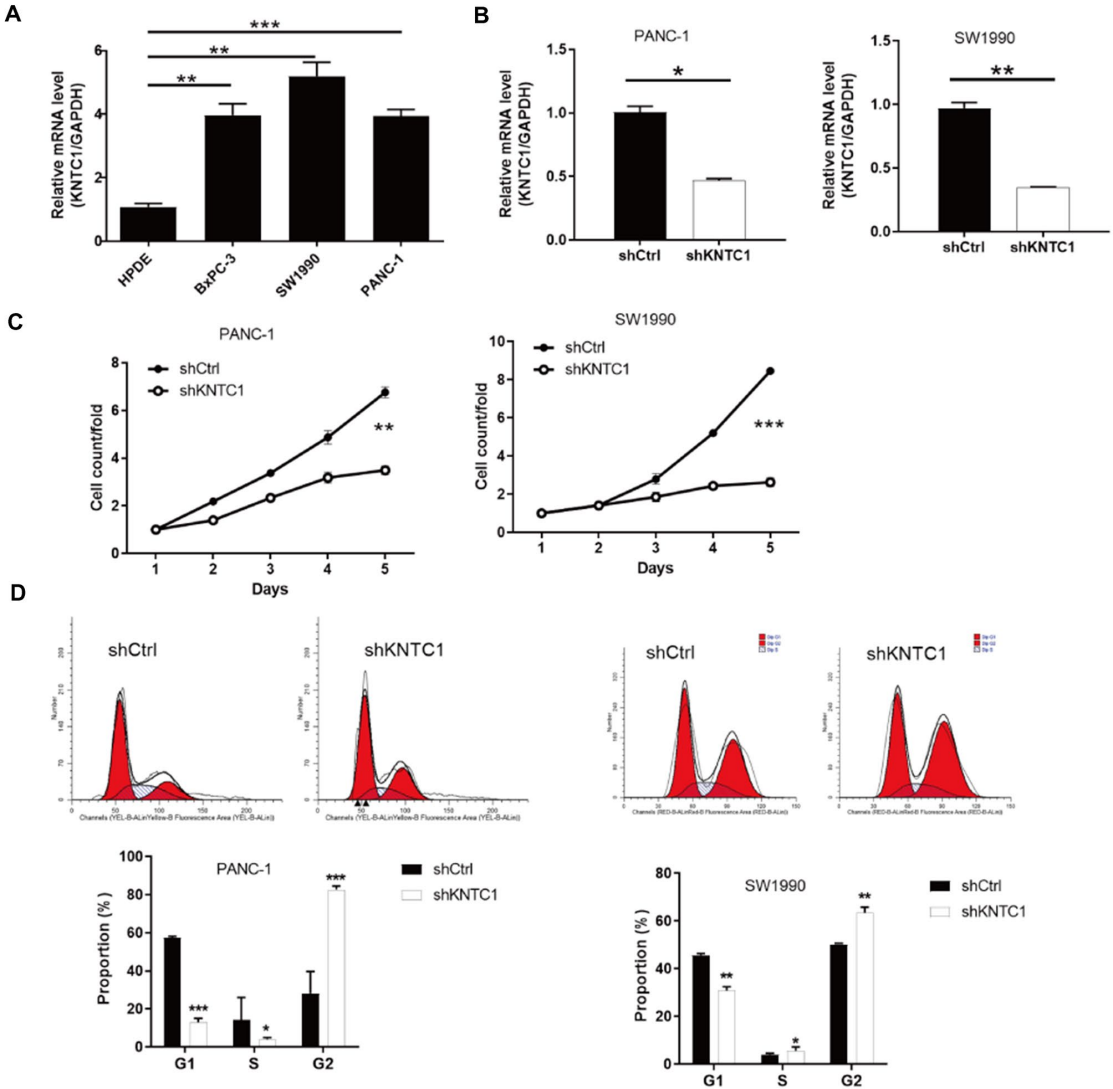
mRNA expression level of KNTC1 in HPDE, BxPC-3, PANC-1, and SW1990 cells (**A**). lentivirus-mediated shRNA targeting KNTC1 was introduced into PANC-1 and SW1990 cells (**B**). The CCK8 assay of KNTC1 knockdown in PANC-1 and SW1990 cells (**C**). The level of apoptosis of KNTC1 knockdown using annexin V staining using flow cytometry (**D**)

We determined the expression levels of KNTC1 in three human pancreatic cancer cell lines (BxPC-3, PANC-1, and SW1990). We found that KNTC1 expression levels were significantly upregulated in the pancreatic cancer cell lines compared to normal human pancreatic ductal epithelial (HPDE) cells. To explore the role of KNTC1 knockdown in the biological behavior of pancreatic cancer cells, lentivirus-mediated shRNA targeting KNTC1 was introduced into PANC-1 and SW1990 cells. We evaluated cell proliferation using the CCK8 assay, which showed that KNTC1 knockdown significantly inhibited the viability of PANC-1 and SW1990 cells. To confirm that apoptosis was induced by KNTC1 knockdown, we performed annexin V staining using Flow cytometry. The level of apoptosis in the KNTC1 group was higher than that in the sham group (Fig. 3). Therefore, knockdown of KNTC1 can inhibit the proliferation of pancreatic cancer cells and block the cell cycle in the G2 phase.

### Association between KNTC1 expression and function in pancreatic cancer

**Fig. 4:**
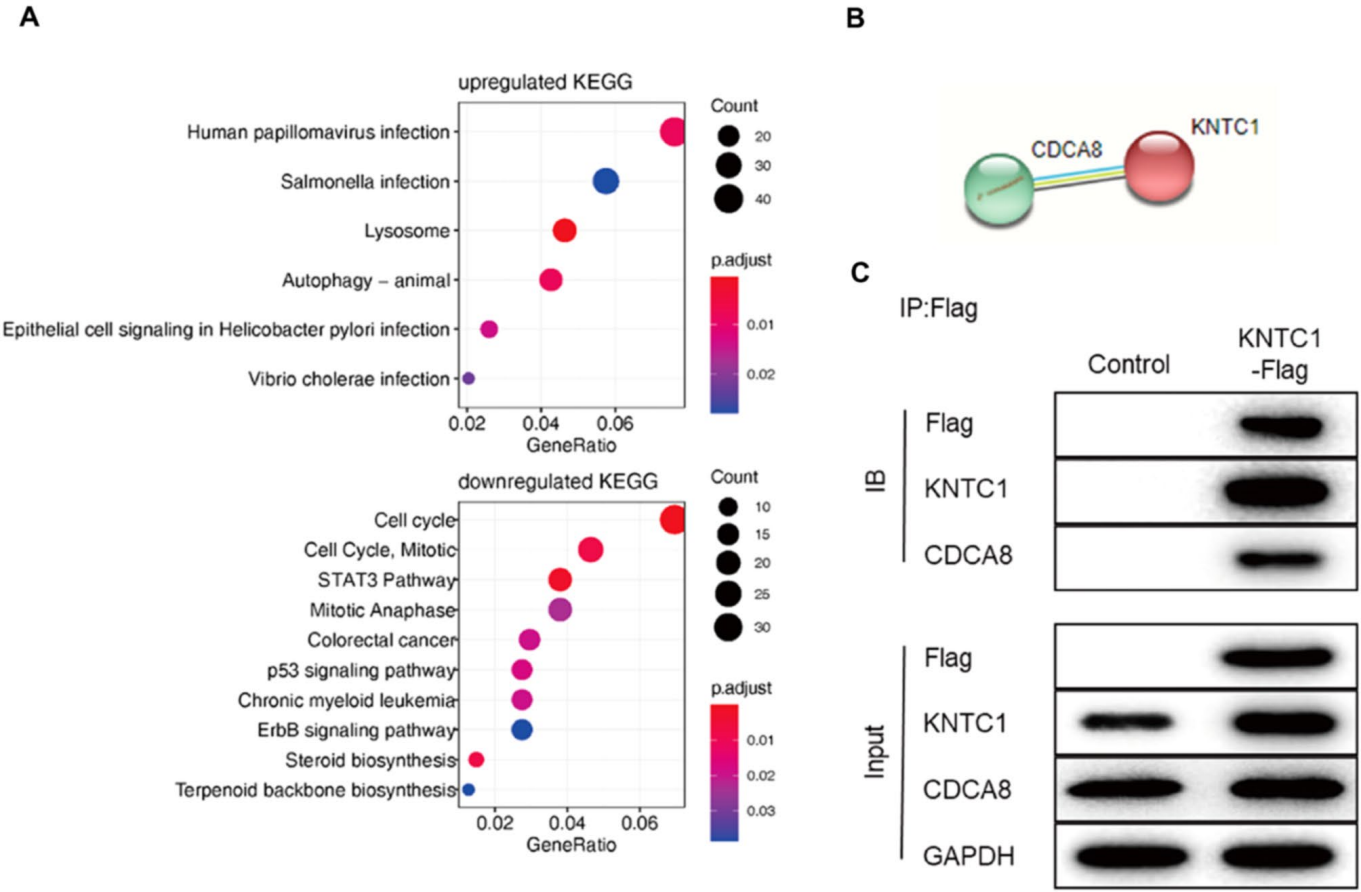
Classical signal pathway enrichment of GO, KEGG, and co-expression gene analysis of KNTC1 in pancreatic cancer (**A**). The interaction between KNTC1 and CDCA8 by immunoprecipitation (**B**, **C**)

We performed transcriptome sequencing on the total RNA obtained from shKNTC1 (KNTC1 knockout cells) and control groups. Classical signal pathway enrichment of GO, KEGG, and co-expression gene analysis were performed. Bioinformatic analysis showed that the differentially expressed genes were mainly concentrated in the cell cycle, mitosis, and STAT3 signaling pathways. We confirmed the interaction between KNTC1 and cell division cycle associated 8 (CDCA8) by immunoprecipitation, which indicates that KNTC1 interacts with CDCA8 in pancreatic cancer (Fig. 4).

## Discussion

Cell division is a series of precisely ordered processes that are regulated by several mechanisms, such as cyclins and cyclin-dependent kinases (Canman and Cabernard 2018). If the regulation mechanism is abnormal, it can have catastrophic consequences. One of the remarkable characteristics of tumor cells is the emergence of aneuploid inheritance and malignant proliferation. The KNTC1 protein is a subunit of the outer structure of the centromere, which plays a key role in the regulation of the spindle checkpoint (Wang et al. 2021; Li et al. 2016). Checkpoint devices produce inhibitory signals during mitosis, delaying the process from metaphase to anaphase so cells can accurately connect the centromere and microtubules. If the checkpoint mechanism is abnormal, aneuploid cells appear. The three activities that have been ascribed to KNTC1 are: (1) recruitment of cytoplasmic dynein to the kinetochore, (2) participation in the poleward movement of chromosomes during mitosis, and (3) maintaining a functional metaphase checkpoint. However, little is understood of the function of KNTC1 in pancreatic cancer (Liu et al. 2019a).

We found, by RNA sequencing combined with differential gene analysis, that the expression of KNTC1 was upregulated in pancreatic cancer. According to the TCGA database, high expression levels of KNTC1 are related to poor prognosis. Further research determined that the high expression of KNTC1 in pancreatic cancer was related to the pathological grade and clinical prognosis of pancreatic cancer, indicating that the expression of KNTC1 was higher in pancreatic cancers with a higher pathological grade and later stage. Similarly, we found that the expression of KNTC1 was higher in three groups of pancreatic cancer cell lines (BxPC-3, PANC-1, and SW1990) than in normal pancreatic ductal cells. We also found that KNTC1–shRNA-transduced cells exhibited reduced cell growth and viability compared to the control group. In addition, KNTC1 knockout increased apoptosis in PANC-1 and SW1990 cells. Bioinformatic analysis of KNTC1-knockout cells showed that differential genes were mainly enriched in the cell cycle, mitosis, and STAT3 signaling pathways, and co-immunoprecipitation confirmed the interaction between KNTC1 and CDCA8.

CDCA8, which is regulated by the cell cycle, is an important part of the centromere protein complex, which is necessary for chromatin-induced microtubule stability and spindle formation (Qi et al. 2021; Zhang et al. 2015). Its function in the centromere is to ensure the correct arrangement and separation of chromosomes. Thus, CDCA8 is recognized as an important regulator of mitosis and cell division. Several reports have shown that CDCA8 participates in tumor carcinogenesis (Gao et al. 2020; Hayama et al. 2007). Jeon et al. found that silencing CDCA8 can inhibit the growth of HCC by restoring the ATF3 tumor suppressor gene and inactivating the carcinogenic AKT/β-catenin signal. CDCA8 could be a promising molecular target in the treatment and prevention of metastasis or recurrence in primary liver cancer (Cui et al. 2021; Jeon et al. 2021). Therefore, we infer that KNTC1 can bind and interact with CDCA8, causing an increase in unstable mitotic spindles, eventually leading to the occurrence and development of cancer by regulating the cell cycle. The present study is the first to identify the role of KNTC1 in regulating the growth of pancreatic cancer. However, the function of KNTC1 and the key molecular mechanism of pancreatic cancer require further research.

In summary, we found that high expression of KNTC1 in pancreatic cancer was related to pathological grade and clinical prognosis. Knocking down KNTC1 inhibited the growth of pancreatic cancer and promoted apoptosis. Further analysis revealed the interaction of KNTC1 and CDCA8 in the regulation of the cell cycle. However, the specific molecular mechanism remains unclear.
